# The Antioxidant Effect of Colombian Berry (*Vaccinium meridionale* Sw.) Extracts to Prevent Lipid Oxidation during Pork Patties Shelf-Life

**DOI:** 10.3390/antiox10081290

**Published:** 2021-08-14

**Authors:** Márcio Vargas-Ramella, José M. Lorenzo, Sol Zamuz, María Esperanza Valdés, Daniel Moreno, María C. Guamán Balcázar, José M. Fernández-Arias, Jorge F. Reyes, Daniel Franco

**Affiliations:** 1 Centro de Educação Superior da Região Sul—CERES da Universidade do Estado de Santa Catarina, Laguna 88790-000, Brazil; marcio.ramella@hotmail.com; 2 Centro Tecnológico de la Carne de Galicia, Rúa Galicia N° 4, Parque Tecnológico de Galicia, San Cibrao das Viñas, 32900 Ourense, Spain; jmlorenzo@ceteca.net (J.M.L.); solzamuz@ceteca.net (S.Z.); 3 Área de Tecnología de los Alimentos, Facultad de Ciencias de Ourense, Universidad de Vigo, 32004 Ourense, Spain; 4 Centro de Investigaciones Científicas y Tecnológicas de Extremadura (CICYTEX), Instituto Tecnológico Agroalimentario de Extremadura (INTAEX), Av. Adolfo Suárez s/n, 06007 Badajoz, Spain; esperanza.valdes@juntaex.es (M.E.V.); daniel.moreno@juntaex.es (D.M.); 5 Departamento de Química, Universidad Técnica Particular de Loja, San Cayetano Alto s/n, Loja 1101608, Ecuador; mcguaman@utpl.edu.ec (M.C.G.B.); jmfernandez@utpl.edu.ec (J.M.F.-A.); jfreyes@utpl.edu.ec (J.F.R.)

**Keywords:** lipid oxidation, phenolic compound, pork patties, natural antioxidant, sensory analysis, *Vaccinium meridionale* Sw.

## Abstract

A scarce amount of knowledge about the use of Colombian berry (CB) in meat products is available in the literature. This work studies the impact of the addition of CB extracts (CBE) on pork patties at three different concentrations in the range 250–750 mg/kg. CBE were characterized in terms of their polyphenolic profile and antioxidant activity [1,1-diphenyl-2-picrylhydrazyl (DPPH) radical scavenging capacity, half maximal inhibitory antioxidant concentration (IC_50_), 2,2′-azino-bis-3-ethylbenzothiazoline-6-sulfonic acid (ABTS), ferric reducing antioxidant power assay (FRAP) and oxygen radical absorbance capacity (ORAC) tests)]. After pork patties elaboration, instrumental and sensorial colour, as well as lipid oxidation measured as thiobarbituric acid reactive substances assay (TBARS) values, were evaluated for 10 days of refrigerated storage in a modified atmosphere (80% O_2_–20% CO_2_). The total anthocyanin composition represented 35% of the polyphenolic substances of the CBE, highlighting high contents in cyanidin derivatives. Additionally, other flavonoids (quercetin and kaempferol compounds) and phenolics acids, substances positively related to antioxidant activity, were identified and quantified. In addition, the incorporation of CBE resulted in improvements in colour and lipid stability of pork patties, especially for the highest concentration used. Our findings demonstrated that CBE could be added to pork patties without impairing their sensorial profile. Overall, our results indicate that the use of CBE as a source of natural antioxidant, natural colourant, or even as a functional ingredient could be promising, but more studies are necessary to confirm it.

## 1. Introduction

Ground meat products (e.g., patties, burgers) are widely acknowledged around the world due to their ease of preparation and consumption in terms of cooking time, as well as their nutritive inherent value [1,2]. However, despite this fact, these meat products have high water content and low levels of antioxidant compounds, hence they are prone to lipid oxidation [3]. It is well established that food oxidation processes can promote the degradation of fat-soluble vitamins and essential fatty acids as well as generate potentially harmful compounds [4]. Furthermore, several studies have identified that meat patties oxidize more rapidly than whole cuts as grinding disintegrates muscular membranes, releasing compounds that favour reactions among prooxidant molecules and unsaturated compounds [2]. Therefore, processing methods of pork patties make them liable to develop oxidative rancidity during elaboration and storage, resulting in an important cause of quality deterioration (e.g., flavour, colour, and texture) [5]. From these meat quality attributes, colour is the most important since it is an indicator of freshness and has a crucial influence on the consumer purchase decision. Specifically in meat products, the oxidation of heme proteins results in a deterioration of meat colour during storage [6]. Consequently, these changes in organoleptic attributes may result in shelf life reduction, consumer dissatisfaction, and product rejection [1].

In this context, the usage of synthetic antioxidants is one of the major approaches for preventing these oxidative reactions and extend the meat products shelf life. However, these additives have various negative healthy connotations [7]. Considering this, in recent years, researchers and the meat industry have focused on natural ingredients from plants and fruits as an alternative to synthetic antioxidants [8,9,10,11]. Furthermore, ascorbic acid is widely used in the food industry for its antioxidant and stabilising ability [10]; although it is naturally present in fruits and some vegetables, it is economically obtained by chemical synthesis for its use as an additive. Those extracts from plants and fruits (seeds, peels, barks, woods, flowers, and leaves) that are recognized as GRAS (generally recognized as safe) have a broad acceptance from consumers [2] and usually, they are rich in phenolic compounds which have important antioxidant and antimicrobial activity [3,12,13,14,15].

Concerning their application in meat products, a large variety of fruit extracts have been reported to be effective in the improvement of the meat oxidative stability, highlighting berries especially for the phenolic compounds and vitamins (C and E) as reviewed by Lorenzo et al. [16]. Wild bilberries are known for their bioactive properties and their antioxidant capacity, attributed to their high content and variety of polyphenolic compounds [12,17]. Within berries, the genus *Vaccinium* comprises a group of plants that includes up to 450 species and the berries of many of them are widely consumed by humans. In particular, the Colombian berry (*Vaccinium meridionale* Sw.), also known as Colombian bilberry, “agraz”, Andean berry, or “mortiño”, is one of the species that grow in the Andean region of South America. Garzón et al. [18] reported that this fruit has high antioxidant activity and potential applications as a source of phytochemicals in the nutraceutical and functional food market because it is an excellent source of dietary phytochemicals such as polyphenolics compounds. More recently, several studies have been conducted to determine the phenolic profile of Colombian berry [19,20], as well as the application of Colombian berry extract (CBE) as an antioxidant in beef patties [21]. However, the assessment of CBE as a natural antioxidant in meat products is still scarce. Considering that pork is the most widely eaten meat in the world (>36% of the world intake) [22] and that patties are popular products in the fast-food industry, stores, restaurants, and households [2] with a limited shelf-life, it would be of interest to assess the CBE as an antioxidant.

Therefore, the present study aimed to investigate the effects of the addition of a CBE, its physicochemical (pH, colour, and lipid oxidation) characteristics, and the sensory quality of pork patties during nine days of refrigerated storage. Moreover, the phenolic profile and antioxidant capacity of the CBE have been carried out.

## 2. Materials and Methods

### 2.1. Plant Material

The fruits (*V. meridionale* Sw.), were collected from Cañi (Tangabana of Chimborazo province, Ecuador). The fruit was washed and disinfected with a chlorine solution (100 ppm). Berry processing was carried out in a brand pulper (KitchenAid^R^, St. Joseph, MI, USA) with a mesh size of 2 mm to obtain the by-product of *V. meridionale* Sw.

### 2.2. Colombian Berry (V. meridionale Sw.) Extraction Procedures—Preparation of Ethanolic Extract from Colombian Berry (CBE)

Colombian berries (*V. meridionale* Sw.) were dried at 40 °C for 12 h in a tray dryer (DY-110H, Daeyeong E&B CO., Ltd., Ansan, South Korea). Samples were ground, sifted, and homogenized (particle size range <350 µm) before extraction and were stored at refrigerated conditions until the process was carried out. Extraction conditions were carried out as follows: a solid (berry) to liquid (solvent) ratio of 1:5 (g/mL) was used, as well as ethanol/water (50:50 *v*/*v*) for three days at refrigerated temperature. Afterwards, the ethanolic Colombian berry extract was evaporated in a rotavapor (G1, Heidolph, Schwabach, Germany) at 40 °C and 150 rpm. Finally, the extract was frozen at −40 °C and lyophilized in freeze-drying (Labconco, Kansas City, MO, USA) with the following settings (pressure = 0.180 mbar; temperature = −50 °C) for 48 h. After freeze-drying, the Colombian berry extract obtained (CBE) was added to pork patties in different doses, as described in the Section 2.4.

#### 2.2.1. In Vitro Antioxidant Activity of CBE

To assess the antioxidant activity of the CBE by four different tests, the lyophilized extract was dissolved in methanol in a ratio solid (mg): liquid (mL) of 3:1 for 30 min at room temperature. The 1,1-diphenyl-2-picrylhydrazyl (DPPH) radical scavenging capacity was determined in CBE using 2 mL of a 6 × 10^−5^ M methanolic solution of DPPH, which was mixed with 50 μL of a methanolic solution of the lyophilized extract. The inhibition percentage (IP) was calculated as the percentage reduction in absorbance at 515 nm after 16 min to the initial value and the half maximal inhibitory antioxidant concentration (IC_50_) as the concentration of extract required to quench 50% of the initial DPPH radical. The results for DPPH and IC_50_ were expressed as µg of Trolox equivalents (TE)/g and mg/mL, respectively.

The 2,2′-Azino-Bis-3-Ethylbenzothiazoline-6-Sulfonic Acid (ABTS^●^^+^) assay was performed following the protocol described by Re et al. [23]. This method is based on the scavenging of ABTS cation radical observed as a decolourization of the blue-green colour at 734 nm. Solutions of 7 mM ABTS^●^^+^ and 2.45 mM potassium persulfate in phosphate buffer saline (PBS, pH 7.4) stood overnight to generate the ABTS^●^^+^ radical cation (ABTS^●^^+^). Then, the ABTS^●^^+^ stock solution was diluted with PBS and equilibrated at 30 °C to an absorbance of 0.8–0.9 at 734 nm (PerkinElmer^®^ Lambda 25 UV/Vis spectrophotometer, PerkinElmer Inc., Massachusetts, MA, USA). Triplicate determinations were performed by mixing 100 μL of methanolic solution of the lyophilized extract with 1.9 mL of radical solution. The decline in absorbance was followed for 10 min. Appropriate solvent blanks were run for each sample. The radical scavenging capacity was compared with that of Trolox; these results are expressed as g of ascorbic acid per kg of extract.

The oxygen radical absorbance capacity (ORAC) assay was realized following the procedure described previously by Quintero-Quiroz et al. [20]. 2,2′-Azobis-(2-amidinopropane)-dihydrochloride (AAPH) was used as the peroxyl radical generator, Trolox as the standard, and fluorescein as the fluorescent probe. Fluorescein, AAPH, and samples were prepared in a 75-mmol buffer at pH 7.4. The methanolic solution of the lyophilized extract (3 mg/mL) or Trolox standards (25 μL) was mixed with 150 μL of 1 μmol/L fluorescein and pre-incubated at 37 °C for 30 min before 25 μL of AAPH solution (200 mmol/L) was added. The fluorescence at an excitation wavelength of 485 nm and an emission wavelength of 520 nm was measured every 2 min for 120 min using a Spectra Max Gemini EM (Molecular Devices, Orleans Dr, Sunnyvale, CA, USA). A calibration curve with Trolox (12.5–100 μmol/L) was performed for quantification purposes. The results are expressed as mg of Trolox equivalents (TE) per g of sample.

The ferric reducing antioxidant power (FRAP) reagent was freshly prepared with 25 mL of 300 mmol/L acetate buffer (pH 3.6), 2.5 mL of a 10 mmol/L solution of 2, 4, 6-tripyridyl-s-triazine in 40 mmol/L HCl, and 2.5 mL of 20 mmol/L FeCl_3_·6H_2_O in distilled water. This solution was used as a blank, and a solution of 1 mM ascorbic acid was used for calibration. Three mL of the FRAP reagent was mixed with 100 μL of extracts, and the absorbance was monitored at 593 nm. FeSO_4_ aqueous solutions were used for calibration. Reducing power was determined in 1 mL of methanolic solution of the lyophilized extract which was mixed with 2.5 mL of 0.2 M phosphate buffer (pH 6.6) and 2.5 mL of 1.0% potassium ferricyanide. The mixture was incubated at 50 °C for 30 min and, after the addition of 2.5 mL of 10% trichloroacetic acid, it was centrifuged. Then, 2.5 mL of the supernatant was mixed with 2.5 mL of water and 0.5 mL of 0.1% ferric chloride, before reading the absorbance at 700 nm. The activity was expressed as mol ferric sulphate (Fe^2+^) per kg.

#### 2.2.2. Characterization of Colombian Berry (*V. meridionale* Sw.) Extracts: Total Phenolic Content (TPC), Total Anthocyanin Content (TAC), and Phenolic Profile

To investigate the CBE polyphenolic profile, 25 mL of methanol/formic acid (95:5 *v*/*v*) solution was added to 5 g of the lyophilized CBE. The mixture was sonicated for 20 s at room temperature (Reax control, Heidolph Instruments, Schwabach, Germany) and kept in the dark for 72 h. Upon centrifugation (14,000 rpm at 4 °C for 10 min), the supernatant with polyphenolic substances was collected and stored in the fridge until the analysis.

The total polyphenol content (TPC) in the methanolic acid extract was determined according to Singleton and Rossi [24] and the total anthocyanin (TAC) content was quantified by the pH differential method [25]. These determinations were carried out using an autoanalyzer (Y15, Biosystems, Barcelona, Spain). Total phenolics and total anthocyanin were estimated as mg of gallic acid equivalents (GAE) and malvidin-3-glycoside chloride, respectively.

High performance liquid chromatography (HPLC) separation, identification, and quantification of polyphenols substances were performed on an Agilent 1200 LC system (Agilent Technologies, Palo Alto, CA, USA), equipped with a degasser, quaternary pump, column oven, 1290 infinity autosampler, UV-Vis diode-array detector (DAD), fluorescence spectrophotometer detector (FLD), and the Chemstation software package for LC 3D systems (Agilent Technologies, Palo Alto, CA, USA) to control the instrument and for data acquisition and analysis. Separation was performed in a Licrospher^®^ 100 RP-18 reversed-phase column (250 × 4.0 mm; 5 µm packing; Agilent Technologies, Palo Alto, CA, USA) with pre-column Licrospher^®^ 100 RP-18 (4 × 4 mm; 5 µm packing; Agilent Technologies, Palo Alto, CA, USA). The analysis was carried out as described in Portu et al. [26] with slight modifications to improve peak resolution. For the analysis of anthocyanins, 10 µL of previously diluted (1:25) and filtered (Chromafil PET 20/25, Machery-Nagel, Düren, Germany) extract was injected directly into the HPLC and the column was maintained at 40 °C. For the analysis of non-anthocyanin phenolic compounds, previously isolated according to the methodology described by Castillo-Muñoz et al. [27], 10 µL of filtered (Chromafil PET 20/25, Machery-Nagel, Düren, Germany) extract was injected into the HPLC. For identification and quantification of compounds, chromatograms were recorded at 280, 320, 360, and 520 nm in the DAD detector. Elution order, retention time (RT), UV/vis spectra, and cross-comparison with available standards and with earlier papers [18] on the polyphenolic composition of the fruit of *V. meridionale* Sw. were used to characterize phenolic compounds in the extract. For quantification, DAD chromatograms were extracted at 520 nm (anthocyanins), 360 nm (flavonols), 320 nm (hydroxybenzoic and hydroxycinnamic acids and stilbenes), and 280 nm (flavonols), and the calibration curve of the respective standards (*R*^2^ > 0.999) was used.

### 2.3. Sensory Analysis

The sensory tests for the evaluation of sensory acceptance of patties with different CBE treatments were conducted in the sensory laboratory of the Meat Technology Centre of Galicia (Ourense, Spain). Tests were held in closed individual booths according to ISO 8589:2010/A1:2014 regulation [28], under white light for the attributes evaluated in raw samples and red light for the attributes evaluated in cooked patties. The sensory sessions were carried out at 1, 3, 6, and 9 days of patties elaboration to assess their sensory evolution in raw samples. In addition, on day 1, odour, texture, and taste were studied in cooked samples in an oven at 180 °C until an internal temperature of 70 °C. Samples were offered in disposable plastic dishes presented to the taster coded with a 3-digit number drawn from a table of random numbers [29]. Water and unsalted toasted bread were used at the beginning of sessions and among samples to clean the palate and remove residual flavours. Sensory analysis was conducted with 51 consumers (27 females and 24 males aged from 25–40 years), which were selected based on their availability for the evaluation and interest to participate in the research. They were informed about the objectives of the study and the instructions to complete tests by a trained interviewer before starting. The study was approved by the local committee of Centro Tecnológico de la Carne (SEN/2021).

An acceptance test was applied for all treatments to determine how the consumers liked or disliked the patties. At the initial point (day 1), the tasters were asked to evaluate the sensory attributes in either raw patties (appearance and odour) and cooked patties (odour, texture and taste), as well as their overall acceptance using a 7-points hedonic scale (1 = I dislike very much and 7 = I like very much), according to Meilgaard et al. [30]. During shelf life (days 1, 3, 6, and 9), visual attributes acceptance for red colour and surface discolouration were evaluated using a 5-point hedonic scale (1 = not acceptable, 5 = excellent). Thus, either consumer also evaluated the intensity of these attributes using a lineal structured scale from 0 (minimum attribute intensity) to 10 (maximum attribute intensity).

### 2.4. Experimental Design and Manufacture of the Pork Patties

Five batches (15 units per batch, 3 per sampled point) of pork patties were elaborated in the pilot plant of the Meat Technology Centre of Galicia as follows: (i) control without the addition of antioxidant (CON), (ii) control with ascorbic acid (ASC) (500 mg/kg), and three different treatments with CBE with (iii) 250 mg/kg (CBE250), (iv) 500 mg/kg (CBE500), and (v) 750 mg/kg (CBE750). Patties of 80 g were manufactured using pork lean and backfat from Celta pig breed with an 8% fat content. Lean and backfat were ground through a mincing plate with an 8-mm diameter in a refrigerated mincer machine (La Minerva, Bologna, Italy). Then, in a vacuum maceration tumbler (Fuerpla, Valencia, Spain), minced lean and backfat were mixed for 3 min with 18 g of NaCl per kg, water (10%), and ascorbic acid or CBE for each treatment. Meat mass mixed were maintained at 3 °C for 4 h and then patties were formed in a burger-maker machine (Gaser, A-2000, Girona, Spain).

Patties were packed in 300-mm thick polystyrene trays, which were sealed with polyethylene film 74-mm thick and permeability of 2 mL/(m^2^ bar day) suitable for gas mixtures (VIDUCA, Alicante, Spain). The packaging was carried out using a heat sealer LARI3/Pn T-VG-R-SKIN (Ca.Ve.Co., Palazzolo, Italy). The composition of the modified atmosphere was 80% O_2_–20% CO_2_. To simulate supermarket conditions, the trays were stored at 2 ± 1 °C under light with lux values (digital lux meter, HT 306, HTC Instruments, Faenza, Italy) in the range of 15–20. The light source was conventional, hence, UV was not filtered.

A characterization of meat mass was carried out before the elaboration of the different patties batches following the methodology described by Pateiro et al. [31]. Briefly, the chemical composition in percentage was as follows: moisture (70.06 ± 1.57%), protein (18.52 ± 0.42%), intramuscular fat (8.85 ± 1.71%), ash (1.85 ± 0.10%), and cholesterol (24.39 ± 5.36 mg cholesterol/100 g pork patty). Concerning fat profile, monounsaturated fatty acids (MUFA) were the predominant ones, with percentages of 54.32 ± 0.95%, followed by saturated fatty acids (SFA) (33.80 ± 1.01%), and polyunsaturated fatty acids (PUFA) (11.88 ± 0.20%), with oleic acid (44.70 ± 0.82%), palmitic acid (21.63 ± 0.58%), and linoleic acid (9.34 ± 0.16%) being the most predominant fatty acids in MUFA, SFA, and PUFA, respectively. Percentages of n-3 and n-6 fatty acids in the mixture were 0.94.70 ± 0.04% and 10.94 ± 0.19%, respectively. Lastly, patties were characterized by a cooking loss of 30.47 ± 1.80% and a hardness of 120.45 ± 13.87 N.

### 2.5. Physical Parameters (pH, Colour, and Lipid Oxidation) of the Pork Patties

Analyses were carried out at 1 (24 h after preparing the patties), 3, 6, and 9 days of storage to determine pH, colour, and lipid oxidation (TBARS). The studied parameters were assessed in triplicate for every sampling point. The pH of the samples was measured using a digital portable pH-meter (HI 99163, Hanna Instruments, Eibar, Spain), equipped with a penetration probe. Colour parameters were measured using a portable colourimeter (Konica Minolta CM-600d, Osaka, Japan) with pulsed xenon arc lamp, D_65_ illuminant, 10° viewing angle geometry, and 8 mm aperture size, to estimate the colour of the patties in the CIELAB space: lightness (L*), redness (a*), and yellowness (b*). Chroma (saturation index; C*) and hue (h*) were calculated following equations provided by American Meat Science Association (AMSA) [32]. For each sample, the colour was measured in three homogeneous different points free of fat. The numerical total colour differences (ΔE_s_ and ΔE_t_) [33] were calculated among samples without antioxidant (CON) and treated with the extract (CBE: 250, 500, and 750) (ΔE_s_) and between initial (i: day 1) and final (f: days, 3, 6, and 9) times of storage (ΔE_s_ and ΔE_t_), respectively, as follows:

ΔE_s_ = [(L_CBE_ − L_CON_)^2^ + (a_CBE_ − a_CON_)^2^ + (b_CBE_ − b_CON_)^2^]^1/2^ (1)

ΔE_t_ = [(L_f_ − L_i_)^2^ + (a_f_ − a_i_)^2^ + (b_f_ − b_i_)^2^]^1/2^ (2)

Lipid oxidation was evaluated through TBARs index, which was measured using 2 g of pork patties dispersed in 5% trichloroacetic acid (10 mL) and homogenized in an Ultra-Turrax (Ika T25 basic, Staufen, Germany) for 2 min. The homogenate was maintained at −10 °C for 10 min and centrifuged at 2360× *g* for 10 min. The supernatant was filtered through a Whatman N° 1 filter paper (GE Healthcare Europe, Barcelona, Spain). Then, 5 mL of the obtained supernatant was mixed with 0.02 M thiobarbituric acid (TBA) solution (5 mL) and incubated in a water bath at 96 °C for 40 min. The absorbance was measured at 532 nm. TBA reactive substances (TBARs) values were calculated from a standard curve of malonaldehyde with 1,1-3,3-tetraethoxypropane (TEP) and expressed as an mg MDA/kg sample.

### 2.6. Statistical Analysis

For the statistical analysis of the results, an analysis of variance (ANOVA) using the general linear model (GLM) was utilized for variables of the study. The least-squares mean (LSM) were separated using Duncan’s *t*-test. All statistical tests of LSM were performed for a significance level at *p* < 0.05. Correlations between instrumentally measured colour parameters and visual attributes scores (*p* < 0.05) were determined by correlation analyses using Pearson’s linear correlation coefficient. Univariate linear regression was performed with the variable with the highest correlation, using the sensorial score as the dependent variable and the selected variable as independent [34]. Statistical analysis was performed using the IBM SPSS Statistics 23.0 program (IBM Corp., New York, NY, USA).

## 3. Results

### 3.1. Total Phenolic Content (TPC), Total Anthocyanin Content (TAC), and Phenolic Profile of Colombian Berry (V. meridionale Sw.) Extract (CBE)

Table 1 shows the values of total polyphenol (TPC) and total anthocyanin (TAC) values found in the CBE, in the raw material, and the fresh fruit expressed in mg/kg of berry. The CBE had a TPC of 83976.25 ± 167.90 mg/kg, while the raw material and fresh fruit had 3081.60 ± 6.16 mg/kg and 1151.65 ± 2.3 mg/kg, respectively. Concerning TAC, the contents were 29077.50 ± 747.77 mg/kg, 1067.03 ± 27.44 mg/kg, and 397.7 ± 10.3 mg/kg in the same materials. Thus, the anthocyanin compounds account for 35% of the polyphenolic substances of the CBE.

**Figure 1:**
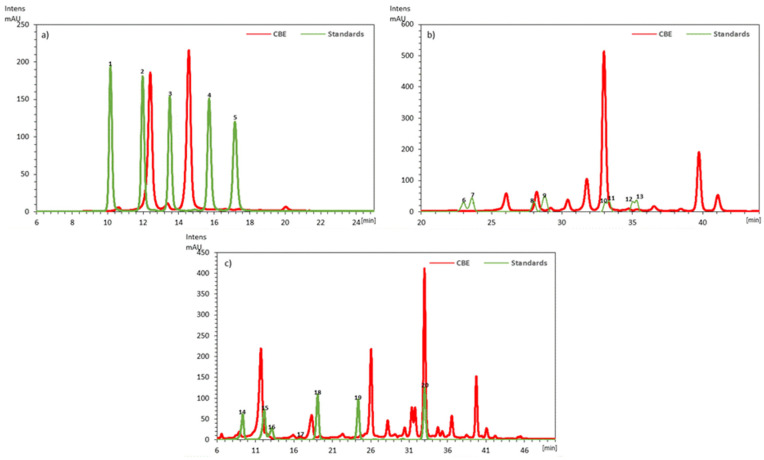
Expanded DAD-chromatograms of CBE and standards for (**a**) anthocyanins (detection at 520 nm), (**b**) flavonols (detection at 360 nm), and (**c**) phenolic acids and stilbenes (detection at 320 nm). (1) Delphinidin-3-glucoside; (2) cyanidin-3-glucoside; (3) petunidin-3-glucoside; (4) peonidin-3-glucoside; (5) malvidin-3-glucoside; (6) myricetin-3-glucoside; (7) myricetin-3-glalactoside; (8) quercetin-3-glucoside; (9) quercetin-3-glucuronide; (10) kaempherol-3-glucoside; (11) kaempherol-3-rutinoside; (12) isorhamnetin-3-glucoside; (13) isorhamnetin-3-rutinoside; (14) *t*-coumaric acid; (15) caffeic acid; (16) syringic acid; (17) fertaric acid; (18) *p*-coumaric acid; (19) ferulic acid; and (20) *t*-resveratrol.

From the anthocyanidins profile of CBE investigated (Figure 1), the most abundant anthocyanidin were the cyanidin derivatives (7729.38 ± 52.15 mg/kg CBE). This amount represents 27% of the total anthocyanins contained in the extract. The HPLC profile of flavanol compounds (Figure 1b) indicated the presence of quercetin (glucuronide and glucoside) and kaempferol (glucoside and rutinoside) as the most abundant compounds of this family. The amount of glucuronide formed in the CBE (982.50 ± 11.30 mg/kg) was higher than that found for glucoside (66.50 ± 3.20 mg/kg CBE). The kaempferol compounds reached the means values of 4460.13, 163.67, and 61.67 mg/kg in the CBE, raw material, and fresh fruit, respectively. Concerning the non-flavonoids compounds, phenolic acids were also identified and quantified. The sum of the content of hydroxycinnamic acids (syringic and p-coumaric) was 98.09, 4.50, and 1.34 mg/kg in the CBE, raw material, and fresh fruit, respectively.

### 3.2. Antioxidant Activity of Colombian Berry (V. meridionale Sw.) Extract (CBE)

The in vitro antioxidant activity of the CBE was evaluated before adding it to pork patties. Results for the four in vitro tests (DPPH, ABTS^●^^+^, ORAC, and FRAP), as well as the IC_50_ using the DPPH procedure, are shown in Table 2.

### 3.3. Evaluation of pH and Colour of Pork Patties during Storage

The effect of the CBE treatment on the pH values, instrumentally measured colour parameters (L*, a*, b*), C*, h*, and ΔE_s_ during the shelf life of patties is summarized in Table 3. The addition of CBE had a significant (*p* < 0.05) effect on the pH and colour values of the patties, except for a* and C* on day 3 (among treatments), b* during storage (CON, ASC, and CBE250 treatments), and ΔE_s_ on days 1, 3, and 6 (among treatments). The highest (*p* ≤ 0.001) pH values were observed in CBE500 and CBE750 in comparison to the other batches (CON, ASC, and CBE250) at the beginning of the experiment (day 1). Afterwards, the pH values of all treatments decreased (*p* ≤ 0.001) with the storage until the sixth day with a slight increase (*p* ≤ 0.001) on the ninth day (except for ASC and CBE750). Indeed, for CBE500 no differences in pH between days 1 and 9 were observed.

Concerning colour parameters, at the beginning of the storage (day 1), CBE500 and CBE750 treatments showed the lowest (*p* ≤ 0.01) L*, while CBE750 had the higher (*p* < 0.05) a*, lower (*p* < 0.001) b*, and lower (*p* ≤ 0.001) h*. On the other hand, at the end of the storage, the CBE750 treatment presented the lowest L* (CBE750 < CBE500 < CB 250, ASC, and CON) and b*, and the highest ΔE_s_ (CBE750 > CBE500 > CB 250). However, despite the extract addition affecting (*p* < 0.05) the patties colour parameters, no significant differences for L*, a*, b*, C*, and h* were observed between CBE250 and CON at day 9.

The a* parameters of patties were in the range of 11.76 (ASC) to 15.67 (CBE750) at the beginning of the storage (day 1), showing a significant (*p* ≤ 0.001) redness decrease at the end of the studied storage (day 9), with values in the range between 2.03 (CON) and 4.44 (CBE750). As seen from Table 3, despite CON and CBE750 batches presenting no significant (*p* ≥ 0.05) differences in a* at day 1, the highest a* values at day 9 were detected in patties with the higher CBE content (CB 500 and CBE750).

Concerning the ΔEs parameter, which indicates the numerical difference among the CON batch and the experimental batches in terms of colour, the values for CBE250, CBE500, and CBE750 until day 6 were in the range between 8.19 (day 1) and 10.3 (day 6), and without significant differences. On the contrary, on day 9, the three CBE batches showed significant (*p* ≤ 0.01) differences among treatments (CBE750 > CBE500 > CBE250), reaching the CBE750 batch the highest ΔEs value.

### 3.4. Evaluation of Lipid Oxidation (TBARS) and ΔE_t_ of Pork Patties during Storage

**Figure 2:**
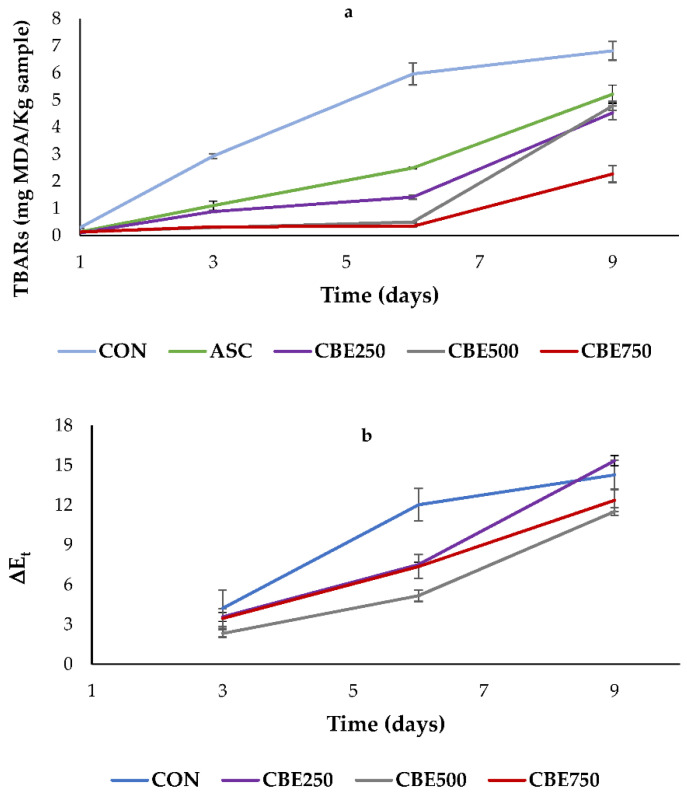
(**a**) TBARS and (**b**) ΔE_t_ values of pork patties during refrigerated storage. Pork patties without antioxidant (CON), treated with ascorbic acid (ASC), and treated with different levels of Colombian berry extract (CBE) (CBE250: 250 ppm; CBE500: 500 ppm; CBE750: 750 ppm).

The results for lipid oxidation and ΔE_t_ during storage are shown in Figure 2. At the beginning (day 1), TBARS values ranged from 0.10 mg MDA/kg (CBE250) to 0.29 MDA/kg (CON), with significant (*p* ≤ 0.01) differences comparing the control with the other batches (CON > ASC, CBE250, CBE500, CBE750). No significant (*p* ≥ 0.05) differences were observed when comparing batches ASC vs. CBE250 and CBE500 at the beginning (day 1; 0.13 vs. 0.10 and 0.14 MDA/kg, respectively) and at the end (day 9; 5.22 vs. 4.53 and 4.78 MDA/kg, respectively) of the storage time. However, the trend showed that, as the days of the storage progressed (days 3 and 6), the patties with the lowest TBARS values were those elaborated with CBE. Moreover, at the end of the refrigerated period, the CBE750 batch displayed the lowest (*p* ≤ 0.001) TBARS value (2.27 MDA/kg; Figure 2a).

It is well-known that lipid oxidation and plant extracts could modify the colour of the patties, hence the total colour difference (ΔE_t_) between days (1 vs. 3, 1 vs. 6, and 1 vs. 9) for four batches (CON, CBE250, CBE500, and CBE750) was calculated. Only significant (*p* ≤ 0.01) differences were observed on day 1 vs. 6 (CON > CBE250, CBE500, and CBE750). On the other hand, colour change considering days 1 vs. 3 and 1 vs. 9 was similar (*p* ≥ 0.05) among samples, indicating an attenuation of the colour modification promoted by CBE treatment at the beginning and the end of the experiment. In addition, in the present study, a positive Pearson’s correlation was found between TBARS values with L* (*R*^2^ = 0.6831, *p* < 0.001; *n* = 60) and h* (*R*^2^ = 0.7833, *p* < 0.001; *n* = 60) and negative between TBARS and a* (*R*^2^ = 0.6770, *p* < 0.001; *n* = 60).

### 3.5. Sensory Analysis

#### 3.5.1. Sensory Acceptance

**Figure 3:**
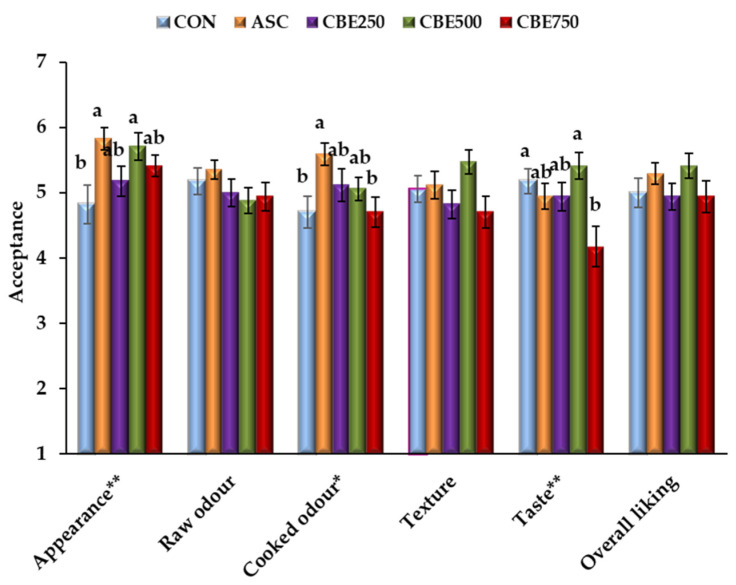
Sensory scores of acceptances attributed by the consumers on day 1 for raw and cooked patties produced without antioxidant (CON), with ascorbic acid (ASC), and with Colombian berry extract (CBE250: 250 ppm; CBE500: 500 ppm; CBE750: 750 ppm). Hedonic scale in either raw patties (appearance and odour) and cooked burgers (odour, texture, and taste), and their overall liking using a 7-points hedonic scale (1 = I dislike very much and 7 = I like very much). Mean values (corresponding to the same parameter) followed by a different letter (a–b) differ significantly (*p* < 0.05; Tukey′s test). * *p* < 0.5; ** *p* < 0.01. Error bars corresponding to standard error.

To determine if the CBE could affect the sensorial characteristics of the pork patties, an acceptance test was conducted (Figure 3). From the test performed, the raw patties showed a significant difference only for appearance (*p* ≤ 0.01), while cooked patties displayed significant differences for odour (*p* < 0.05) and taste (*p* ≤ 0.01). All treatments received an overall liking score between 4.2 and 5.4 (acceptability limit of 3) with no significant (*p* ≥ 0.05) differences among batches, indicating that the addition of CBE did not alter the patties’ acceptability. Therefore, CBE could be used as an ingredient in pork patties without impairing the sensory characteristics of the product.

#### 3.5.2. Visual Attributes Evaluation during Shelf-Life: Red Colour Intensity (RCI) and Surface Discolouration Intensity (SDI)

**Figure 4:**
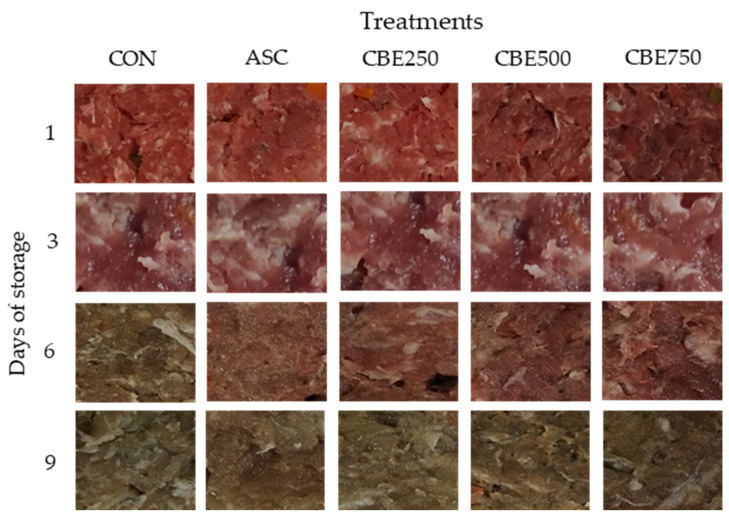
Photographs of pork patties without antioxidant (CON), treated with ascorbic acid (ASC) and treated with different levels of Colombian berry extract (CBE) (CBE250: 250 ppm; CBE500: 500 ppm; CBE750: 750 ppm) in each day of evaluation.

The appearance of the pork patties, assessed visually during the 9 days of storage, is represented in Figure 4. Regarding the evolution of the sensory properties, the addition of CBE affected the colour at day 1 and during the storage period.

**Figure 5:**
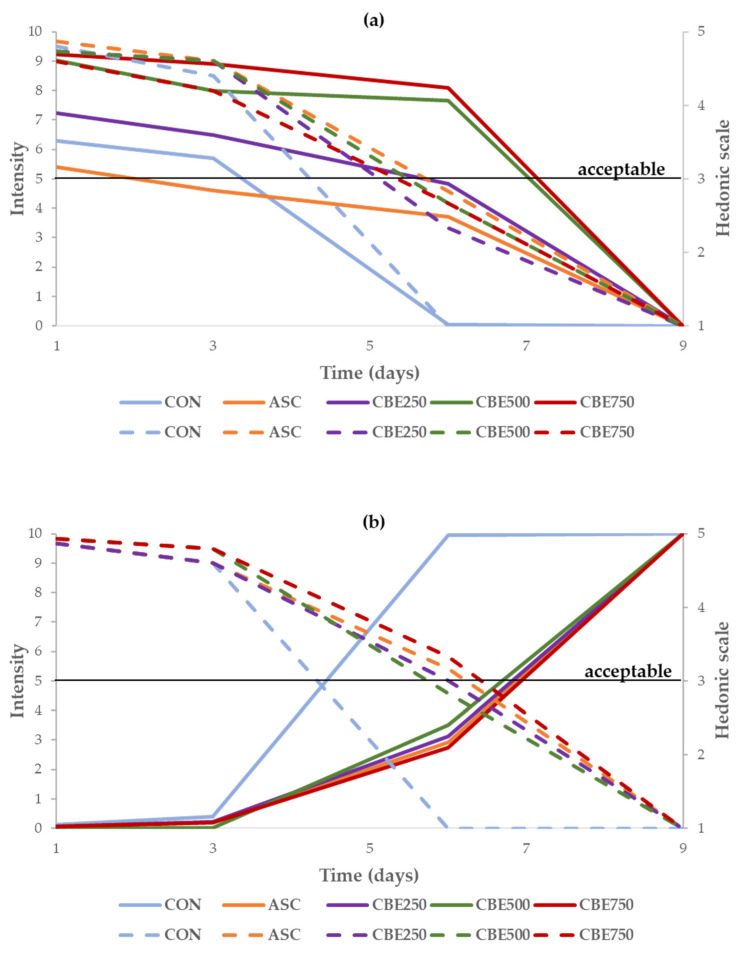
Visual attributes (**a**) red colour and (**b**) surface discolouration evaluated by the consumers during shelf-life (days 1, 3, 6, and 9) for raw patties. CON: without antioxidant; ASC: with ascorbic acid; CBE250: 250 ppm of Colombian berry extract; CBE500: 500 ppm of Colombian berry extract; CBE750: 750 ppm of Colombian berry extract. Intensity of the attributes was evaluated using a linear structured scale from 0 (minimum attribute intensity) to 10 (maximum attribute intensity). Acceptance was evaluated using a five-point hedonic scale (1 = not acceptable, 5 = excellent).

For the analysis of RCI undertaken on the raw patties (Figure 5a), panellists attributed the lower values for CON (6.6) and ASC (5.8) on day 1, while the higher scores were obtained for patties elaborated with Colombian berry extract (CBE250, CBE500, and CBE750; 7.6, 9.5, and 9.4, respectively). On the contrary, only patties with the higher extract amount (CBE500 and CBE750) maintained a higher intensity of red colour until day 6 (RCI ≥ 7.7). For the same period (day 6) CON, ASC, and CBE250 batches received 0.0, 3.7, and 4.8, respectively. Conversely, concerning the hedonic scale, all treatments had similar acceptance values (4.2–4.6) on day 3, meanwhile all batches were considered unacceptable (RCI < 3) on day 6.

Concerning SDI (Figure 5b), all treatments received similar evaluation (0.0–0.4) by consumers until day 3. In addition, on day 6, except for CON (SDI = 10), all treatments were evaluated with lower values for discolouration (SDI ≤ 3.5). Regarding the hedonic scale, CON and CBE500 treatments were already unacceptable (SDI < 3) on day 6, while ASC, CBE250, and CBE750 were still considered acceptable during this period. 

#### 3.5.3. Correlations between Colour Parameters and Visual Attributes of Pork Patties

Given that the red colour parameters during the shelf life of pork patties were extended mostly in the CBE treatment (CBE500 and CBE750 batches showed the higher a* values at day 9), it is interesting to correlate sensory (visual colour assessment scores) and instrumental parameters for these data. Pearson’s correlation coefficients obtained for instrumentally measured colour parameters (L*, C*, and h*) and those from the sensory analysis for visual attributes RCI, SDI, and red colour acceptance (RCA) are presented in Table 4. Regression analysis showed that the correlations were significant (*p* ≤ 0.001) and strong regardless of h* and RCI (−0.947), SDI (0.958), and RCA (−0.932).

**Figure 6:**
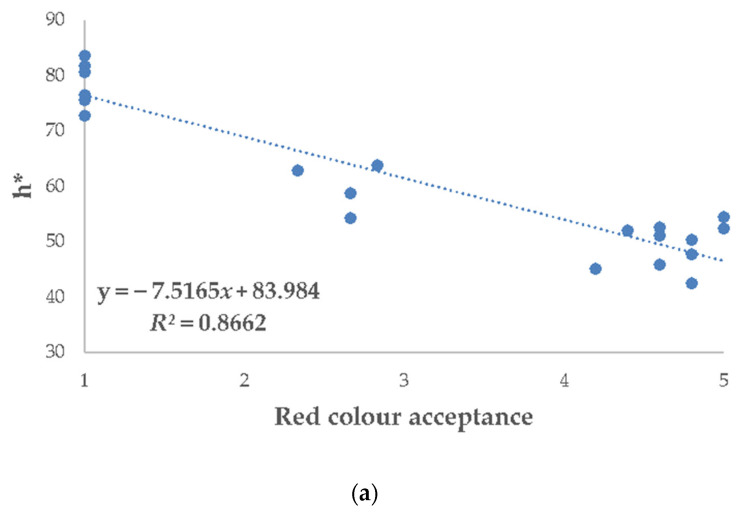

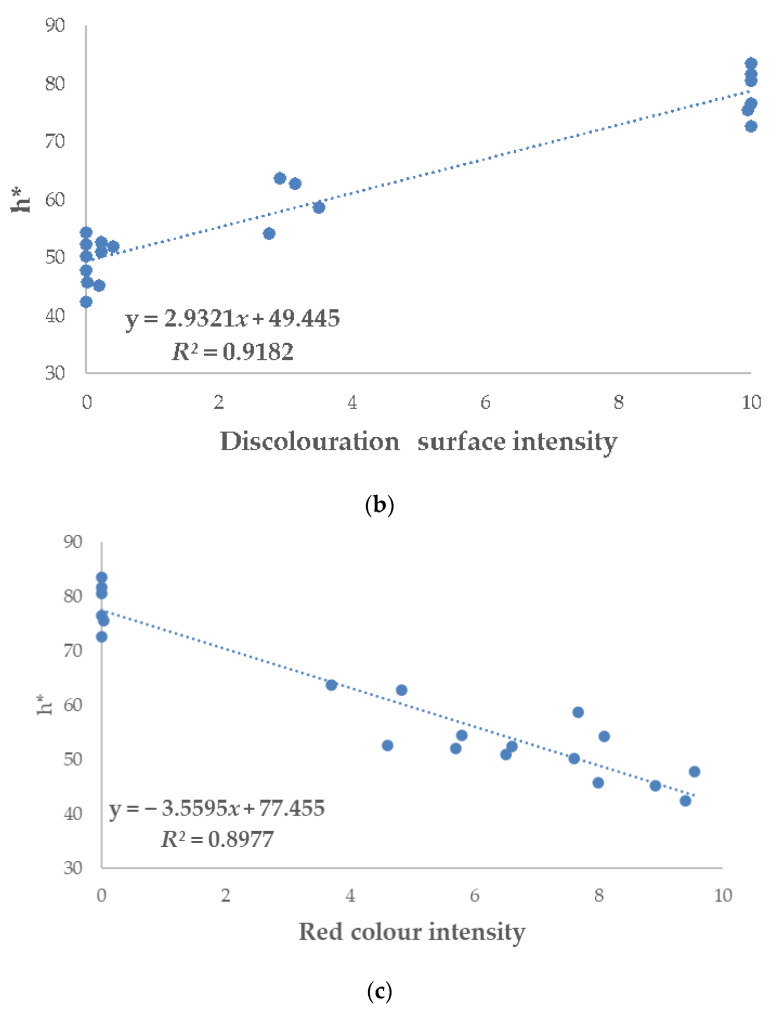
(**a**) Relationship between h* and red colour acceptance; (**b**) Relationship between h* and red colour intensity; (**c**) Relationship between h* and discolouration surface intensity.

Considering that colour parameters were more closely correlated among the h* values and the visual attributes RCI, SDI, and RCA, a linear regression was determined among them (Figure 6). As seen in Figure 5, consumer evaluation for RCA (*R*^2^ = 0.8662) (Figure 6a) and RCI (*R*^2^ = 0.8977) (Figure 6b) linearly increased with the h* decrease. On the contrary, concerning SDI values (Figure 6c), measurements were positively correlated, and consumer scores attributed to SDI (*R*^2^ = 0.9182) increased linearly with h*. Our results demonstrated that h* can reflect consumers colour evaluation for RCI, SDI, and RCA.

## 4. Discussion

### 4.1. Total Phenolic Content (TPC), Total Anthocyanin (TAC), and Phenolic Profile of Colombian Berry (V. meridionale Sw.) Extract (CBE)

The previous studies that identified and quantified the phenolic compounds in fresh fruit, pomace, and other fruits derivatives [18,19,20] concluded that, in the *V. meridionale* Sw., the content of these compounds was quite high compared to other *Vaccinium* species. Thus, it can be considered as a promissory fruit to be employed in the food industry. However, the possible use of Colombian berry as an ingredient in food preparations has still been scarcely studied. In this sense, the identification and quantification of the polyphenolic profile of their extracts from fruits or by-products as a source of nutraceuticals or natural additives are of paramount importance to determine and optimize their further utilization.

The 83,976.25 mg GAE/kg of TPC achieved in the CBE and elaborated in the present study is lower than that reported by Quintero-Quiroz et al. [20] in a freeze-dried extract made from this fruit (139,290 mg GAE/kg). The extracts elaborated from this fruit by López-Padilla et al. [21] by ultrasound-assisted extraction and pressurized liquid extraction had TPC contents between 71.70 ± 6.7 mg GAE/kg and 9.26 ± 2.52 mg GAE/kg. Different and fundamental aspects have to be considered to explain these differences. On the one hand, as it is widely reported for other fruits, it is evident that the methodology used for the preparation has a great influence on the release of polyphenolic substances from the raw material [18].

Moreover, it is evident that the initial TPC of that raw material also has a great effect, as does the fact that the content and type of polyphenols in seeds, pulp, and peel are different, hence the proportion in which these parts are in the raw material has to be considered. Indeed, plant extracts are richer sources of TPC than plant compounds (e.g., seeds, peels, flowers, or leaves) [3,8,35] and their use are generally preferred since are needed in small quantities to be effective [3]. For instance, in the studies of the Garzon group [18,19], the differences in TPC content (pomace > whole fruit) obtained were attributed to the presence/absence of seeds and peels, which contain a larger amount of phenolic compounds, especially anthocyanins and procyanidins [18]. Additionally, it has also been reported that plant phenolics show qualitative and quantitative variation at genetic levels, physiological, and developmental stages of the plant. Phenolics also vary in response to environmental factors, such as light intensity and nutrient availability [12]. All these factors may contribute to the differences in phenolic profiles reported in the literature.

For comparative purposes with different tropical fruits, our TPC values were higher than those published for red pitaya (*Hylocereus monacanthus* Lm. Britton & Rose) (2681.3 mg of GAE/kg extract) [5], Cupuaçu (*Theobroma grandiflorum* (Willd. ex Spreng.) K. Schum.) (4970 mg GAE/kg of fresh weight), Algarrobo (*Hymenaea courbaril* L.) (20130 mg GAE/kg of fresh weight), and Cashew (*Anacardium occidentale* L.) (48,510 mg GAE/kg of fresh weight) [36]. On the other hand, CBE showed lower TPC than those reported for plant extracts from grape seed (*Vitis vinifera* L. and *Vitis labrusca* L.) (373,000 mg GAE/kg), tea (*Camellia sinensis* (L.) Kuntze) (390,900 (mg GAE/kg) [37], and guarana seeds (*Paullinia cupana* K.) (258,000 mg/kg) [38]. 

The content of total anthocyanin (Table 1) found in the CBE, raw material, and fresh fruit of the Colombian berry (*V. meridionale* Sw.) is within the range cited in the previous works. Garzón et al. [18], Maldonado-Celis et al. [39], and Quintero-Quiroz et al. [20] reported means values of 3.29, 1.51, and 4.66 (mg cyanidin equivalents/g) for the freeze-dried Colombian berry values, respectively. Concerning fresh fruit, values of mg cyanidin-3-glycoside/100 g in the range 92–235 mg for the northern highbush blueberry (*Vaccinium corymbosum* L.), 60–187 for the rabbiteye blueberry (*Vaccinium ashei* R.), and 290–300 for lowbush blueberry (*Vaccinium angustifolium* S.) have been reported by several authors [40,41,42,43].

The polyphenolic profile of CBE analysed is quite similar to that found by Vasco et al. [44] for a variety of fresh fruit mortiño (*Vaccinium floribundum* K.) from Ecuador and to those found by Garzón et al. [18,19] for fresh fruits and pomace (*V. meridionale* Sw.) from Colombia. These authors published information concerning phenolic profiles where anthocyanidin compounds (mostly cyanidin and delphinidin derivatives), flavanols (predominantly quercetin), and phenolic acids were the most abundant compounds. Figure 1a shows the peaks that correspond to cyanidin and delphinidin.

The presence of anthocyanins (in general) and cyanidin derivatives (in particular) are important for the antioxidant properties of CBE. Ivanovic et al. [45] found a strong linear correlation between DPPH radical scavenging activity and the content of cyanidin in extracts of blackberry cultivar “Cacanska Bestrna” cultivated in Serbia. Flavonols such as quercetin, kaempferol, and myricetin and their derivatives (primarily glycosides) are considered the dominant flavonoids in bilberries, cranberries, and lingonberries [46]. Quercetin and kaempferol derivatives were identified and quantified in the CBE (Figure 1b). The presence of quercetin derivatives was already reported by Garzon in berries and pomace from *V. meridionale* Sw. [18,19]. Although kaempferol derivatives were not reported in these studies, these flavonoids compounds have been identified in *Vaccinium myrtillus* L. fresh fruits and extracts [47] and *Vaccinium Vitis-idaea* L. fresh fruits [48]. The presence of these compounds is a key factor in the nutraceutical properties of CBE. Quercetin is considered as an exceptional free radical scavenger and consequently, it is suggested to be involved in human health benefits such as cardioprotective effects and chemo-preventive effects against certain types of tumours [49]. Quercetin inhibits human platelet aggregation in vitro by inhibiting protein-tyrosine kinase [50]. Kaempferol is known for its cytotoxic, antioxidant, and apoptotic effects [51].

In our attempt to identify the individual profile of phenolic acids of CBE, we were only able to identify and quantify the p-coumaric and syringic acid. Other identified non-flavonoid compounds (Figure 1) were below the quantification limit. The values found represented a little percentage (<1%) of the total polyphenolic amount of CBE. In this regard, we have to take into consideration the small amounts found in the raw material, and in the fresh fruits lower than 99.2 ± 6.7 mg/100 g of fresh weight reported by Garzon et al. [18]. Unlike our results, some references [18,19] showed the chlorogenic acid and caffeic acid derivatives as those more common non-flavonoid compounds in pomace and berries from *V. meridionale*. For the interpretation of these results, the amount variation of caffeic, ferulic, p-coumaric, chlorogenic acids, and the total phenolic acids in the different *Vaccinium* cultivars [18,44,52,53], should be considered. It is also well known that the extraction method can influence determination of phenolic acids, and their concentration can vary due to different factors as environmental conditions and ripening berries. Therefore, due to the antioxidant compounds assessed in the present study for the CBE, results suggested that the incorporation of this extract in the meat products (e.g., pork patties) could increase their shelf life and improve their nutritional properties.

### 4.2. In Vitro Antioxidant Activity of Colombian Berry (V. meridionale Sw.) Extract (CBE)

DPPH, IC_50_, ABTS^●^^+^, ORAC, and FRAP assays were used to evaluate the in vitro antioxidant activity of CBE. DPPH results and the scavenging effects on DPPH measured as IC_50_ showed values of 143.68 ± 3.87 µg TE/g and 1.55 ± 0.01 mg/mL, respectively. Our results were lower than those obtained by Fernandes et al. [54] who assessed the antioxidant capacity by DPPH tests of 13 plant extracts, obtaining values in the range 5000 to 90,600 µg TE/g of dry weight. These differences can be explained, in part, by the fact that our data are expressed on a wet basis. Concerning IC_50_, our findings showed lower values (higher antioxidant activity) than those reported for brown seaweed extracts (*Fucus vesiculosus* L.) (3.47 mg/mL) [1] and Duzhong extract (*Eucommia ulmoides* Oliv.) (2.81 and 8.43 mg/mL, roasted cortex and seed, respectively) [55]. On the contrary, our results were higher (lower antioxidant activity) than those found for plant origin extracts such as chestnut (*Castanea sativa* Mill.) (0.34 mg/mL) [56], grape seed (*Vitis vinifera* L. and *Vitis labrusca* L.) (0.16 mg/mL), and tea (*Camellia sinensis (L.) Kuntze*) (0.12 mg/mL) [37]. Usually, low values of DPPH scavenging tend to provide higher values for its IC_50_, which indicates lower radical-scavenging activity [1]. Despite that, in the present study, lower values of DPPH scavenging were found when compared to several plant extracts [54], which does not mean necessarily lower antioxidant activity.

Many factors can influence the composition of a plant extract and, consequently, their antioxidant activity assays (e.g., extraction procedures) [57]. Contreras-Calderón et al. [36] reported that FRAP, ABTS^●^^+^, DPPH, and ORAC are the most widely used methods to assess the antioxidant activity of the extracts. Indeed, they recommended at least two of these tests combined to provide reliable information. For this reason, in the present study, different methodologies have been employed to evaluate the in vitro antioxidant capacity of CBE.

The ABTS^●^^+^ method measures the ability of the antioxidant molecule to quench ABTS^●^^+^ radicals via an electron transfer reaction, assessing a radical discolouration [18]. A value of 253.27 g ascorbic acid/kg indicates that CBE can quench ABTS^●^^+^ as an electron donor. This antioxidant capacity could be attributed to a higher level of extracted non-polar phenolic compounds and oxygenated monoterpenes in CBE that could act as direct free-radical scavengers [2,18]. In several studies, authors have reported ABTS^●^^+^ values for CBE of 1.034 mol TE/kg [21], CB fresh fruit (327.9 μmol TE/g), CB fresh weight (45.5 mol TE/g) [18], and CB dry weight (546.7 μmol TE/g) [19]. Moreover, values for ABTS^●^^+^ of guarana seed (*Paullinia cupana* K.) (2072 μmol TE/g) [38], grape seed (*Vitis vinifera* L. and *Vitis labrusca* L.) (2.93 g TE/g), and tea (*Camellia sinensis* (L.) Kuntze) (4.06 (g TE/g) [37] have been published.

Compared to other methods, the ORAC test has received extensive coverage and utilization in the field of antioxidants. The change in fluorescence intensity is an index of the degree of free radical damage. The inhibition of free radical damage by an antioxidant is a measure of its antioxidant capacity against the free radical [58]. Values of ORAC for the CBE of the present study (472.25 mg TE/g) were higher than those previously published for CB pomace extract (416.8 TE/g of dry weight) [19] and freeze-dried CB (159.48 mg TE/g) [20].

The FRAP assay measures the potential of an antioxidant to reduce the yellow ferric-2,4,6-tri-pyridyl-s-triazine (TPTZ) complex to a blue ferrous-TPTZ complex by electron-donating molecules under acidic conditions. Concerning FRAP results, the value of 1.98 mol Fe^+2^/kg in the present study was 16-fold higher than 0.116 mol Fe^+2^/kg of fresh weight reported by Garzón et al. [18].

After analysing our findings in terms of in vitro antioxidant activity of CBE and comparing them with other antioxidants extracts from plant origin, it could be hypothesised that CBE could be used to prevent the oxidation of pork patties. This possibility will be evaluated in the next section. In a way, our results are not surprising in agreement with Lorenzo et al. [16], who recently published a review indicating the use of berry extracts as natural antioxidants in meat products. In the same line, López-Padilla et al. [21] also reported that CB is being valorised as raw material for the recovery of rich antioxidants with the application as natural additives in meat products. The DPPH, IC_50_, ABTS^●^^+^, ORAC, and FRAP values obtained for the CBE can be attributed to the presence of phenolics compounds with high antioxidant and radical scavenging activity [18,19], especially anthocyanins (e.g., monoglucosides of cyanidin and delphinidin), hydroxycinnamic acids (e.g., coumaric acid), and flavanols (e.g., quercetin derivatives).

Finally, it is important to remark that different results were obtained by several authors [18,19,21,59,60] for the phenolic profile and antioxidant activity of CB. This is related to the fact that phenolics and their characteristics depend significantly on the material assessed (e.g., raw fruit, pomace or extract) and/or the extraction technique utilized to prepare the samples (e.g., extraction equipment, solvents, and extraction temperatures). In addition, it is well-known that polyphenols have different abilities to scavenge free radicals [61]. For example, for CB, it has previously been published that the antioxidant activity (ABTS^●^^+^) of the raw fruit (0.007 mol TE/kg) is extremely low when compared with its extract obtained with ethanol at 25 °C (0.140 mol TE/kg) or ethanol/water 50:50 at 200 °C (1.034 mol TE/kg) [21]. In line with this, further studies will be necessary with other procedures to ensure a more accurate comparison and to confirm CBE phenolic profile as well its antioxidant activity.

### 4.3. Evaluation of pH and Colour of Pork Patties during Storage

The lower values of the pH of the pork patties (*p* ≤ 0.001) measured in CON, ASC, and CBE250 treatments at day 1 (CBE500 and CBE750 > CON, ASC, and CBE250) suggested that the pH of the extracts could have an important effect on the initial pH of the patties. Similar findings were reported in previous studies with pork patties formulated with grape (*Vitis vinifera* L.), green tea (*Camellia sinensis* (L.) Kuntze) [37], and watermelon (*Citrullus lanatus* (Thunb.) Matsum. y Nakai) rind extracts [3]. However, Peiretti et al. [62] noticed a contrary trend in pork patties elaborated with blueberry pomace, indicating the higher pH values in control patties with ascorbic acid together those patties formulated with a 2% of blueberry pomace, in disagreement with our results. In line with this, other studies with pork patties, enriched with plants extracts such as guarana seeds (*Paullinia cupana* K.) [38], thyme (*Thymus serpyllum* L.) [2], and pitanga leaf (*Eugenia uniflora* L.) [63,64], have described a significantly (*p* ≤ 0.01) initial decrease in the pH. The highest pH values showed in the patties with a higher extract content (CBE500 and CBE750) could be explained by the characteristics of the berry used to obtain the extract, as well as the nature of its polyphenols/active compounds among others.

As expected, the addition of CBE affected all colour parameters which seem to be directly linked with the dark red colour of the extract (Figure 4). The darkening of the pork patties as a consequence of the addition of CBE has also been noticed in other studies with blueberry [62] added to pork patties or with red-blue fruits [65] in the elaboration of chicken meat. With other types of natural extract, such as thyme (*Thymus serpyllum* L.) [2], green tea (*Camellia sinensis* (L.) Kuntze), grape (*Vitis vinifera* L. and *Vitis labrusca* L.), chestnut (*Castanea sativa* Mill.), and seaweed (*Ulva lactuca* L. and *Ulva rigida* C. Agardh) [37], no modification of the meat product lightness has been reported.

Concerning redness index (a*), the lower values observed in the patties with the lowest concentration of CBE (CBE250) or without berry extract (CON and ASC) are previously reported by authors that attributed these reductions to lipid oxidation [2,66]. This redness loss in the patties during the storage is probably associated with the myoglobin oxidation, which is red in its reduced form and brown in the corresponding oxidized ferric form [67]. This discolouration can be alleviated by reducing the formation of metmyoglobin using plant extracts, particularly those rich in monoterpene polyphenols [66,68].

The C* (saturation index) is related to pigment concentration and refers to the colour intensity or its relative strength, and therefore is different to L* which instead measures colour lightness when compared against a white standard. This suggests C* as a better means to account for the qualitative-determined importance of red colour brightness [69]. During storage time and until the 9th day, the C* parameter decreased (*p* ≤ 0.001), but in a different way among treatments. This means that patties throughout storage displayed a less vivid colour and were close to grey.

As the h* parameter is calculated based on a* and b*, a higher h* means the development of colour from red to yellow. Indeed, h* is considered as a measure of colour change noticeable by the human eye and represents colour stability [70]. An increasing trend in the h* values during storage time and until the ninth day was assessed in the present study, the h* parameter raised (*p* ≤ 0.001) indicating gradual oxidation of myoglobin and accumulation of metmyoglobin with time [71]. Therefore, a higher h* detected in CON (CON, CBE250 > CBE500 > CBE750, respectively) would indicate colour development of these patties to a smaller redness index, suggesting higher oxidation during the storage.

The colour difference (ΔE) is described by the National Bureau of Standards Unit, stating that there are small differences among colours that are detectable by humans: 0–0.5 (trace), 0.5–1.5 (slight), 1.5–3.0 (noticeable), 3.0–6.0 (appreciable), 6.0–12.0 (much), >12.0 (very much) [68]. Consequently, according to these standards, in the present study, the colour differences were “much detectable” throughout the storage until day 6 (ΔE_s_ > 6), and in a similar way among the three treatments when compared to CON. On the opposite, on day 9 of storage, all samples showed significant (*p* ≤ 0.01) differences in ΔE_s_ among them, which means that colour difference (ΔE_s_ > 3) could be “appreciable” for each treatment [68]. Our results agree with a previous study with beef patties that evaluated the potential of CB to protect myoglobin, as well as its global contribution to product colour. The findings of the study led to an evident total colour difference between treated and control samples (ΔE_s_) in which the treated samples could be discriminate from the control [21].

Changes in the colour of CON patties were expected since previously it has been reported that, when the patties are kept refrigerated, samples without antioxidants suffer a more intense deterioration of L*, b*, and especially a*values. Indeed, this decreasing tendency is expected and very characteristic in raw meat and meat preparations [21]. However, despite our data suggesting a potentially lower rate of colour deterioration of patties elaborated with CBE, it should be noted that it is not advisable to use a ΔE value as a single way to measure colour deterioration since ΔE involves changes of L*, a*, and b* which do not always change in parallel [68].

### 4.4. Evaluation of Lipid Oxidation and ΔE_t_ of Pork Patties during Storage

Increased lipid oxidation observed in the samples is likely due to endogenous enzymatic activity (e.g., lipoxygenase). Furthermore, the protein degradation process of myoglobin stimulates the release of high concentrations of free iron with high prooxidant potential [2]. Therefore, as predicted, TBARS values were higher in the CON batch, but they significantly (*p* ≤ 0.001) increased in all treatments during the storage, characterizing the progression of lipid oxidation. However, concerning patties with antioxidants (ASC and CBE), only CBE750 treatment maintained TBARS values below 0.5 mg MDA/kg over a storage period (9 days), which is marked as an acceptable sensory limit for revealing rancid flavour [72].

Our results for the initial TBARS values showed no significant (*p* > 0.05) differences among batches, as one might expect. In disagreement, López-Padilla et al. [21] reported significant differences in TBARs values in beef patties elaborated with CBE at day 0. The authors attributed these differences to CBE pigments (authors used 20,000 ppm of CBE) as they are capable to absorb light in the same wavelength range as MDA.

In the present study, although significant differences were observed in colour parameters (Table 3) among treated samples (day 1), this effect was not observed on TBARS values. It can thus be suggested that CBE has an immediate effect on the colour of the patties, especially in L* and a*, previously to preservation exerted by active compounds (polyphenols). This finding has an interesting implication on the use of CBE with a dual purpose (as a natural colourant and as an antioxidant). In the present study, the CBE250 and CBE500 batches had similar values for TBARS when compared to the ASC group. These results showed that CBE might be a potent antioxidant in the control of lipid oxidation during refrigerated storage, as no prooxidant effect attributable to CBE was found in the studied range (250 to 750 ppm). Nonetheless, existent literature on the use of CBE as an antioxidant ingredient in foods is scarce. Further studies evaluating the addition of CBE to other complex meat matrices, such as meat emulsions or meat cooked products, are necessary to corroborate this information.

Even though lower L* and h* and higher a* and b* were obtained for CBE500 and CBE750 treatments in comparison to CON batch, no significant (*p* ≥ 0.05) differences were observed for ΔE_t_ among samples at the end of the experiment (day 1 vs. 9). Thus, except for day 1 vs. 6, no positive effect of CBE on the colour of the patties concerning CON was observed. In addition, all patties (CON and CBE treatment) showed ΔE_t_ values between 2.33–4.22 (day 1 vs. 3) and 11.52–14.28 (day 1 vs. 9), which is over the value of 2, and considered a limit to appreciate visual colour changes [37]. Therefore, results would indicate that consumers will perceive colour changes suffered by patties during storage (days 3, 6, and 9) for each treatment. Finally, the findings of the current study for the TBARS correlation with colour parameters (positive correlation with L* and h* and negative correlation with a*) were partially in agreement with Nassu et al. [71]. They also found an increasing/decreasing trend for lipid oxidation and colours parameters; however, with a negative correlation among TBARS with a∗ and C* and a positive correlation between TBARS and h*. Still, as previously discussed for colour (Section 4.3), ΔE_s_, and lipid oxidation (Section 4.4), it is not advisable to use this parameter as a single way to measure colour deterioration [68].

### 4.5. Sensory Analysis

#### 4.5.1. Sensory Acceptance

The possible benefits of the inclusion of plant extracts on meat products cannot diminish their sensory characteristics, specifically colour. Indeed, consumer acceptability is one of the critical factors for the development of new foodstuffs [56]. In line with this, our outcomes for sensory acceptance (appearance, raw odour, cooked odour, texture, taste, and overall impression) showed scores > 4.2 for all CBE treatments on the hedonic scale (over “acceptable”). This result could be attributed to the CBE phenolic profile. In this regard, Pateiro et al. [35] corroborated those compounds, such as hydroxycinnamic acid, anthocyanins, tannins, and flavonoids, could be responsible for the quality improvement of pork patties, resulting in a product with higher sensory acceptability.

The high liking score results obtained from the hedonic tests are extremely encouraging since this is not always the case in the reformulation and development of functional meat products [61]. As the preference score averages can be considered an appropriate means to determine consumer acceptance [56], our results indicate that CBE could be added as an additive into pork patties without impairing sensory attributes.

#### 4.5.2. Visual Attributes Evaluation during Shelf-Life: Red Colour Intensity (RCI) and Surface Discolouration Intensity (SDI)

The incorporation of CBE into pork patties modified the RCI (CBE250, CBE500, CBE750 > CON, ASC) on day 1 and during the storage until day 6. Conversely, the CBE effect on SDI was comparable to ASC since all treatments, except for CON, showed similar scores for this attribute in the same period. Colombian berries are fruits with a purple-dark colour at maturity [20]. Therefore, the CBE affected the RCI (Figure 4) due to its natural pigments rich in anthocyanins (e.g., monoglycosides of cyanidin and delphinidin) and flavanols (e.g., quercetin derivatives) [18,19]. Although, the effect on the colour indicated that only the highest doses of CBE (CBE500 and CBE750) were effective to remain colour stable (RCI) until day 6.

In addition, visual attributes showed can be associated with the lower h* values determined in CBE pork patties (especially CBE500 and CBE750; Table 3). The effect on h* value of meat products is related to the antioxidative compounds that can preserve a red/purplish colour and prevent SDI on patties, reducing oxidation of Mb (metmyoglobin formation), which is red in its reduced-ferric form and brown in its oxidized-ferric form [66,68,73,74].

This positive effect on colour supports the feasibility of using CBE with a dual purpose (as an antioxidant and natural pigment), avoiding the use of synthetic antioxidants and colourants with this purpose. This result for CBE is encouraging since the consumer´s decision to purchase meat products is greatly influenced by colour [75]. Indeed, consumer preference for meat colour is not sufficient to affect taste ratings but is sufficient to affect their likelihood of purchasing [76]. Consequently, comparing the effect of CBE treatments to ASC on pork patties, it seems that CBE was more effective in maintaining RCI and had a similar effect on SDI. Overall, this study has shown that CBE has the potential as a natural preservative to reduce colour degradation.

#### 4.5.3. Correlations between Colour Measurements and Visual Attributes of Pork Patties

As mentioned previously, colour is the most important quality attribute for consumers since it is an indicator of freshness. Numerous studies evaluating the shelf-life of meat products use instrumental measurements to determine their colour. However, although these variables are very useful to typify this parameter, their use raises doubts about how humans perceive or evaluate meat products. In addition, it is difficult to define when these products will be rejected by consumers [34].

Visual evaluation affords the advantages of being able to make a global appraisal of the colour status of the entire measurement surface rather than having to take readings for only a few individual points. Conversely, the colour comparison standards may be rendered unsuitable or may become hard to apply because of factors, such as differences in lighting (metamerism) [77], availability of trained/untrained panellists, or the lack of ability of the trained panel to detect variations among storage time and treatments [71]. On the other hand, instrumental colour readings, by comparison, offer the advantage of not being subject to variation with changes in lighting or consumers. Putting aside economic and/or availability possibilities, overall, both instrumental and visual colour measurements have advantages and disadvantages, but they are complementary to the interpretation of results, and should be taken whenever possible.

The regression coefficient (*R*^2^ ranging between 0–1) shows the accuracy of a model, whereby if this value is higher than 0.70, it indicates that the model is well adjusted [78]. All the R^2^ values obtained in the present study were above this target value (>0.86). Our data showed that a strong relationship among h*, RCI, SDI, and shelf-life of pork patties exists. This correlation with the instrumental colour measurements corroborates the usefulness of the colour comparison reference standards for its use in visual colour evaluations. At the same time, visual appraisals of colour that were closely correlated with the instrumental were useful to assess the panellist’s accuracy. Finally, our results agree with Wang et al. [79] that have demonstrated that h* measurements could reflect consumers colour evaluation.

## 5. Conclusions

The evidence from this study suggests that CBE has the potential as a novel food additive to prevent lipid oxidation during pork patties’ shelf-life. The CBE displayed greater antioxidant activity in protecting the patties against lipid oxidation during storage, as confirmed by the decrease in TBARS (especially for a dose of 750 mg/kg), which provided a higher extended shelf life until day 6 in comparison to control. In addition, CBE could be advantageously incorporated in pork patties without causing detrimental changes in their sensory properties since this did not alter their overall liking of them. Furthermore, as the extracts reduced the loss of redness by delaying discolouration, our findings support the idea that they could be used as a natural antioxidant in meat products as substitutes for synthetic antioxidants. Further investigations should evaluate the ability of CBE as a potential substitute for curing agents in fermented, cured, and cooked meat products, as well to explore its utilization in nutraceutical, pharmaceutical, and food applications associated with its content in interesting polyphenolic compounds.

## Figures and Tables

**Table 1:** Total and individual content (mg/kg) of phenolic compound/subclass identified in Colombian berry (*V.meridionale* Sw.) methanolic acid extract (MAE), raw material, and fresh fruit.

Substance	Content (mg/kg of Berry) ^1^		
	CBE	Raw Material	Fresh Fruit
Total polyphenolic	83,976.25 ± 167.90	3081.60 ± 6.16	1151.65 ± 2.30
Total anthocyanin	29,077.50 ± 747.77	1067.03 ± 27.44	398.77 ± 10.30
Individual polyphenolic compounds			
Cyanidin derivatives	7729.38 ± 52.15	283.64 ± 1.90	106 ± 0.70
Quercetin-3-glucuronide	982.50 ±11.30	36.05 ± 0.40	13.47 ± 0.20
Quercetin-3-glucoside	66.50 ± 3.20	2.44 ± 0.10	0.91 ±0.0
Syringic acid	60.13 ±1.59	2.21 ±0.0	0.82 ±0.0
p-Coumaric acid	37.96 ±1.78	1.39 ±0.0	0.52 ±0.0
Σ Kaempferol derivatives (3-glucoside + rutinoside)	4460.13 ± 44.7	163.67 ± 1.6	61.17 ± 0.6

^1^ Results are expressed as mean ± SD. CBE: Colombian berry extract.

**Table 2:** In vitro antioxidant activity of Colombian berry (*V. meridionale* Sw.) extract.

DPPH ^1,^*	IC_50_^2,^*	ABTS^●^^+ 3,^*	ORAC ^4,^*	FRAP ^5,^*
143.68 ± 3.87	1.55 ± 0.01	253.27 ± 12.57	472.25 ± 6.08	1.98 ± 0.02

^1^ µg trolox/g, ^2^ mg/mL, ^3^ g ascorbic acid/kg, ^4^ mg TE/g, ^5^ mol ferric sulphate/kg. * Results are expressed as mean ± SD. 1,1-diphenyl-2-picrylhydrazyl (DPPH) radical scavenging capacity, half maximal inhibitory antioxidant concentration (IC_50_), 2,2′-azino-bis-3-ethylbenzothiazoline-6-sulfonic acid (ABTS), ferric reducing antioxidant power assay (FRAP) and oxygen radical absorbance capacity (ORAC).

**Table 3:** Effect of the treatments in the pH and colour parameters (L*, a*, b*, C*, h*, and ΔE_s_) of the pork patties during storage.

Parameters	Day	Treatments					SEM	*p*-Value
		CON	ASC	CBE250	CBE500	CBE750		
pH	1	5.58 ^a,B^	5.57 ^a,B^	5.57 ^a,B^	5.67 ^a,A^	5.68 ^a,A^	0.014	<0.001
	3	5.46 ^b,D^	5.53 ^b,B,C^	5.54 ^a,A,B^	5.49 ^c,C,D^	5.57 ^b,A^	0.012	0.002
	6	5.32 ^c,C^	5.41 ^c,A,B^	5.37 ^c,B,C^	5.42 ^d,A,B^	5.47 ^c,A^	0.016	0.014
	9	5.49 ^b,B^	5.42 ^c,C^	5.43 ^b,C^	5.61 ^b,A^	5.53 ^b,c,B^	0.020	<0.001
SEM		0.029	0.022	0.025	0.030	0.026		
*p*-value		<0.001	<0.001	<0.001	<0.001	0.002		
L*	1	48.90 ^b,A^	46.93 ^b,A,B^	43.32 ^c,B,C^	42.96 ^c,C^	39.75 ^b,C^	0.963	0.002
	3	46.84 ^b,A^	48.06 ^b,A^	46.81 ^b,A^	42.69 ^c,B^	42.28 ^a,b,B^	0.694	0.001
	6	53.98 ^a,A^	50.34 ^b,B^	48.15 ^b,B,C^	45.30 ^b,C,D^	42.15 ^a,b,D^	1.156	<0.001
	9	55.75 ^a,A^	55.50 ^a,A^	54.18 ^a,A^	49.40 ^a,B^	44.86 ^a,C^	1.158	<0.001
SEM		1.264	1.084	1.204	0.821	0.648		
*p*-value		0.009	0.002	<0.001	<0.001	0.016		
a*	1	14.45 ^a,A,B^	11.76 ^a,C^	13.13 ^a,B,C^	13.25 ^b,B,C^	15.67 ^a,A^	0.433	0.019
	3	13.59 ^a^	13.08 ^a^	13.20 ^a^	15.20 ^a^	13.55 ^b^	0.284	0.095
	6	4.42 ^b,C^	8.25 ^b,A,B^	7.58 ^b,B^	8.67 ^c,A,B^	9.06 ^c,A^	0.466	<0.001
	9	2.03 ^c,B^	2.69 ^c,B^	2.42 ^c,B^	3.85 ^d,A^	4.44 ^d,A^	0.261	<0.001
SEM		1.678	1.227	1.359	1.328	1.316		
*p*-value		<0.001	<0.001	<0.001	<0.001	<0.001		
b*	1	18.65 ^A^	16.47 ^B^	15.78 ^B,C^	14.61 ^b,C,D^	14.30 ^a,D^	0.444	<0.001
	3	17.39 ^A^	17.10 ^A,B^	16.28 ^A,B^	15.60 ^a,B^	13.60 ^b,C^	0.402	0.001
	6	17.21 ^A^	16.69 ^A^	14.79 ^B^	14.29 ^b,B^	12.57 ^c,C^	0.463	<0.001
	9	18.12 ^A^	16.24 ^B^	16.75 ^A,B^	16.08 ^a,B^	14.21 ^a,b,C^	0.386	0.004
SEM		0.289	0.233	0.305	0.250	0.222		
*p*-value		0.288	0.662	0.105	0.007	0.001		
C*	1	23.63 ^a,A^	20.25 ^a,b,B^	20.53 ^a,B^	19.73 ^b,B^	21.21 ^a,B^	0.431	0.008
	3	22.08 ^a^	21.53 ^a^	20.96 ^a^	21.78 ^a^	19.20 ^b^	0.363	0.058
	6	17.77 ^b,A^	18.62 ^b,A^	16.64 ^b,B^	16.72 ^c,B^	15.50 ^c,C^	0.304	<0.001
	9	18.23 ^b,A^	16.46 ^c,B,C^	16.93 ^b,A,B^	16.54 ^c,B,C^	14.89 ^c,C^	0.343	0.012
SEM		0.814	0.621	0.641	0.674	0.801		
*p*-value		0.001	0.001	0.001	<0.001	<0.001		
h*	1	52.40 ^c,A^	54.38 ^c,A^	50.24 ^c,A,B^	47.78 ^c,B^	42.40 ^c,C^	1.207	<0.001
	3	51.96 ^c,A^	52.64 ^c,A^	50.96 ^c,A^	45.76 ^c,B^	45.13 ^c,B^	0.909	<0.001
	6	75.56 ^b,A^	63.71 ^b,B^	62.85 ^b,B,C^	58.75 ^b,C^	54.21 ^b,D^	1.967	<0.001
	9	83.60 ^a,A^	80.59 ^a,B^	81.74 ^a,A,B^	76.53 ^a,C^	72.69 ^a,D^	1.100	<0.001
SEM		4.254	3.370	3.874	3.692	3.607		
*p*-value		<0.001	<0.001	<0.001	<0.001	<0.001		
ΔE_s_	1			6.590	7.633	10.360	0.960	0.284
	3			2.9800	4.9100	6.0400	0.698	0.206
	6			7.147	10.230	13.557	1.212	0.072
	9			2.490 ^C^	7.013 ^B^	11.837 ^A^	1.452	0.003

CON: control, ASC: ascorbic; CBE250: Colombian berry extract 250 ppm, CBE500: Colombian berry extract 500 ppm, CBE750: Colombian berry extract 750 ppm. SEM: Standard error mean. Values with different letters (A–D) in the same row are significantly different (*p* < 0.05); values with different letters (a–d) in the same column are significantly different (*p* < 0.05).

**Table 4:** Correlations between instrumental colour parameters (L*, C*, and h*) and the visual attributes (red colour intensity, surface discolouration intensity, and red colour acceptance) were evaluated by panellists in sensory analysis of pork patties.

	L*	C*	h*	RCI	SDI	RCA
L*	1	−0.264	0.851 ***	−0.839 ***	0.707 ***	−0.660 ***
C*		1	−0.662 **	0.522 **	−0.735 ***	0.837 ***
h*			1	−0.947 ***	0.958 ***	−0.932 ***
RCI				1	−0.909 ***	0.851 ***
SDI					1	−0.967 ***
RCA						1

RCI: Red colour intensity; DSI: surface discolouration intensity; RCA: Red colour acceptance. ** *p* < 0.05; *** *p* < 0.01.

## Data Availability

Data is contained within the article.
